# Increased plasma and adipose tissue levels of ANGPTL8/Betatrophin and ANGPTL4 in people with hypertension

**DOI:** 10.1186/s12944-018-0681-0

**Published:** 2018-03-01

**Authors:** Mohamed Abu-Farha, Preethi Cherian, Mohamed G. Qaddoumi, Irina AlKhairi, Devarajan Sriraman, Muath Alanbaei, Jehad Abubaker

**Affiliations:** 1 Biochemistry and Molecular Biology, Dasman Diabetes Institute, Kuwait, Kuwait; 2 Pharmacology and Therapeutics Department, Faculty of Pharmacy, Kuwait University, Kuwait, Kuwait; 3 National Dasman Diabetes Biobank, Dasman Diabetes Institute, Kuwait, Kuwait; 4 Department of Medicine, Faculty of Medicine, Kuwait University, Kuwait, Kuwait

**Keywords:** Hypertension, Angiopoietin-like proteins, Type-2 diabetes

## Abstract

**Background:**

Hypertension is a risk factor for both cardiovascular diseases (CVDs) and type 2 diabetes (T2D). Angiopoietin-like proteins (ANGPTLs), mainly ANGPTL3, ANGPTL4 and ANGPTL8, are associated with increased plasma lipid content due to their role in regulating the activity of lipoprotein lipase, a key enzyme in metabolism of the lipoprotein in circulation. Dyslipidaemia is a risk factor for hypertension development; however, the roles of ANGPTL3, ANGPTL4 and ANGPTL8 in subjects with hypertension have not yet been established. This study compared the plasma and adipose tissue levels of ANGPTL3, ANGPTL4 and ANGPTL8 in age- and body mass index-matched subjects with and without hypertension.

**Methods:**

A total of 119 subjects, including 69 hypertensive and 50 non-hypertensive subjects, were enrolled. ANGPTL3, ANGPTL4 and ANGPTL8 plasma levels were measured by ELISA, whereas their levels in adipose tissue were assessed via real-time PCR.

**Results:**

We found that ANGPTL4 (202.49 ± 17.44 ng/mL vs. 160.64 ± 10.36 ng/mL, *p* = 0.04) and ANGPTL8 levels (2310.96 ± 194.88 pg/mL vs. 1583.35 ± 138.27 pg/mL, *p* = 0.001) were higher in hypertensive subjects than non-hypertensive subjects. However, ANGPTL3 levels were not significantly different between the two populations. Similarly, ANGPTL4 and ANGPTL8 levels were also elevated in subjects with T2D and hypertension than in those with T2D but not hypertension. Additionally, people with highest tertiles of ANGPTL8 had higher odds of having hypertension (odd ratio [OR] = 3.8, 95% confidence interval [CI] = (1.5-9.8), *p*-Value = 0.005. Similar to its plasma levels, ANGPTL4 and ANGPTL8 were higher in adipose tissue.

**Conclusions:**

In conclusion, our data illustrate that ANGPTL4 and ANGPTL8 levels in both plasma and adipose tissues are increased in subjects with hypertension. The elevated levels of ANGPTL4 and ANGPTL8 in hypertensive subjects highlight their potential involvement, their potential role as biomarkers for hypertension and their therapeutic value in hypertension given their roles in regulating lipid metabolism.

## Background

Hypertension is a major risk factor for cardiovascular diseases (CVDs) and type 2 diabetes (T2D) [1]. Hypertension is present in 50%–80% of subjects with T2D, highlighting their common aetiology [2]. Nonetheless, hypertension is a modifiable risk factor that has received tremendous attention regarding efforts to reduce the risks and public health burden of T2D and CVDs. Obesity, dyslipidaemia, inflammation and insulin resistance are common factors that increase the risks of hypertension and T2D. Better understanding of various factors involved in hypertension development will lead to improved early detection and prevention of the onset of hypertension, T2D and CVDs.

We recently illustrated that angiopoietin-like protein 8 (ANGPTL8), also known as betatrophin, is strongly associated with high sensitivity C-reactive protein (HsCRP) levels and an increased incidence of metabolic syndrome [3]. Certain biomarkers such as high-sensitivity C-reactive protein (HsCRP) can act as early signs of increased risks for hypertension and T2D development [4, 5]. Many epidemiological studies have confirmed that high HsCRP levels are associated with increased risks of CVDs and metabolic syndrome [4]. HsCRP can also act as an independent predictor of the development of new-onset hypertension after adjusting for multiple risk factors as shown in the Women’s Health Study [6]. Other cohort studies reported similar data linking CRP levels with hypertension resulting from vascular inflammation or elevated blood pressure that in turn leads to increased vascular inflammation [6]. HsCRP has been shown to interact with oxidized LDL and β2-glycoprotein I forming complexes that promotes atherosclerosis in diabetic mice for example [7, 8]. ANGPTL3, ANGPTL4 and ANGPTL8 regulate the activity of lipoprotein lipase (LPL), a key enzyme in the hydrolysis of plasma lipoproteins [9–11]. Hydrolysis of triglycerides (TGs) in plasma by LPL generates free fatty acids that are taken up by peripheral tissues for storage or energy production [9]. Both ANGPTL3 and ANGPTL4 are well-established inhibitors of the enzymatic activity of LPL under feeding and fasting conditions, respectively [9]. Recently, ANGPTL8 was shown to inhibit LPL activity in the feeding state [9]. In addition, recent studies involving examination of mutations in the ANGPTL4 gene revealed that these mutations were associated with lower plasma lipid levels and reduced cardiovascular risk [12]. Furthermore, variants in the ANGPTL8 gene are associated with lower plasma low-density lipoprotein (LDL) and high-density lipoprotein (HDL) levels in Hispanic and African Americans [11]. Given the role of ANGPTL3, ANGPTL4 and ANGPTL8 in regulating lipid metabolism we hypothesised that their levels might be increased in subjects with hypertension. Therefore, we examined the plasma and adipose tissue levels of ANGPTL3, ANGPTL4 and ANGPTL8 in subjects with or without hypertension using ELISA and real-time PCR.

## Methods

### Study population and ethical statement

In the present study, 119 subjects, including 69 subjects with hypertension and 50 without hypertension, were enrolled. Subjects with hypertension were defined as males and females with systolic blood pressure of ≥140 mmHg and/or diastolic blood pressure of ≥90 mmHg or those using antihypertensive medications. The subjects were age- and body mass index (BMI)-matched between the groups. BMI was calculated using the following standard formula: body weight (in kilograms)/height (in metres squared). The study was approved by the Ethical Review Board of Dasman Diabetes Institute study number (RA2016-025) and conducted in accordance with the Declaration of Helsinki. Approved written informed consent was provided by all subjects before their participation in the study. The exclusion criteria were type 1 diabetes, prior major illness and the use of any medication and/or supplement known to influence body composition or bone masses as well as people enrolled in physical exercise program within the last six months [13].

### Blood collection and anthropometric and biochemical measurements

Blood samples were collected as previously outlined [14, 15]. Briefly, after they signed the consent form, fasting blood samples were collected from the participants into Vacutainer EDTA tubes. Plasma was prepared via centrifugation of the blood-containing tubes at 400×*g* for 10 min, after which it was aliquoted and stored at − 80 °C until analysis [13, 16, 17]. Participants also consented to undergo biopsy of the subcutaneous adipose tissue (SAT) that was obtained from the periumbilical area via surgery after local anaesthesia, as described previously [13]. SAT was rinsed in cold PBS, completely submerged in All Protect Tissue Reagent to stabilise RNA, DNA and protein and then stored frozen at − 80 °C until use. Blood pressure was measured using an Omron HEM-907XL Digital sphygmomanometer. The average value of three blood pressure readings was recorded. Whole-body composition was determined using a dual-energy radiographic absorptiometry device (Lunar DPX, Lunar radiation, Madison, WI). Using a Siemens Dimension RXL chemistry analyser (Diamond Diagnostics, Holliston, MA), fasting blood glucose, TG, total cholesterol, LDL and HDL levels were measured. Glycated haemoglobin content was measured using a Variant™ device (BioRad, Hercules, CA).

### ANGPTL8 Elisa

The circulating level of ANGPTL8 was measured using an ELISA kit, as described previously. Briefly, plasma samples were thawed on ice and centrifuged at 10,000×*g* for 5 min at 4 °C to remove any cells or platelets remaining in the sample [13, 16, 17]. A Wuhan EIAAB Science ELISA kit (catalogue number E1164H) was used to measure ANGPTL8 content, as described previously [16, 18, 19]. No significant cross-reactivity with other proteins was observed. The intra-assay coefficients of variation (CVs) were 3.1%–5.7%, whereas the inter-assay CVs ranged 6.2%–9.8%.

### ANGPTL3 and ANGPTL4 ELISA

Plasma levels of ANGPTL3 and ANGPTL4 were assessed using the multiplexing immunobead array platform according to the manufacturer’s instructions (R&D Systems). The data were processed using Bio-Plex manager software version 6 (BioRad) with five-parametric curve fitting. The intra-plate CV ranged from 6.0% to 13%, whereas the inter-plate CV was < 15%. Samples were measured using reagents from the same batch to avoid inter-batch variations.

### Measurement of gene expression by real-time quantitative PCR

Total RNA was extracted from frozen SAT using an RNeasy Lipid Tissue Mini Kit (Qiagen, Valencia, CA) according to the manufacturer’s protocol. Total RNA was isolated from adipose tissue biopsies of obese non-diabetic (*n* = 8) and obese diabetic subjects (*n* = 8). cDNA was prepared from total RNA samples using High Capacity cDNA Reverse Transcription Kits (Applied Biosystems, Foster City, CA). Real-time quantitative PCR was performed on a Rotor-Disc 100 system using SYBR Green normalised to *Gapdh* (Qiagen). The PCR primers were as follows: ANGPTL3 forward, TCTCCAGAGCCAAAATCAAGAT, reverse, TTTCACTGGTTTGCAGCGAT; ANGPTL4 forward, CAGTCCTCGCACCTGGAA, reverse, GCCAGGACATTCATCTCGTC and ANGPTL8 forward, AATCTGCCTGGATGGAACTG, reverse, CTGCGTCTGTCTCTGCT-CTG. GAPDH was used as a loading control with the following sequences: forward, AACTTTGGCATTGTGGAAGG, reverse, TGTGAGGGAGATGCTCAGTG. Relative gene expression was assessed using the ∆∆CT method [20].

### Statistical analysis

Comparisons between hypertensive and non-hypertensive subjects were made using Student’s *t*-test. Spearman’s correlation coefficients were estimated to determine the associations between ANGPTL8 and ANGPTL3 and ANGPTL4. A multivariable logistic regression analysis was performed to estimate odds ratios (ORs) adjusted for covariates to assess the predictive power of ANGPTL 3, 4 and 8 for hypertension. All data are reported as the mean ± standard deviation. Statistical assessments were two-sided and considered to be significant when *p* value was < 0.05. All analyses were performed using SAS (version 9.r; SAS Institute).

## Results

### Study population characteristics

Our population included 119 subjects with or without hypertension. All subjects were matched for age and BMI between the two groups as shown in Table 1. The mean age of the hypertensive subjects in the whole population was 53.41 ± 11.50 years and that of the non-hypertensive subjects was 53.62 ± 10.80 (*p* = 0.44). The mean BMI was 32.56 ± 3.93 kg/m^2^ for hypertensive subjects and 32.20 ± 4.37 kg/m^2^ for those non-hypertensive subjects (*p* = 0.65). Tables 2 and 3 also show the characteristics of the diabetic and non-diabetics with and without hypertension.

**Table 1:** Characteristics of all subjects included in this study according to their hypertension status

Variables	Hypertension *N* = 69	Non- Hypertension *N* = 50	*p* value
Age (years)	53.41 ± 11.50	53.62 ± 10.80	0.44
BMI (kg/m^2^)	32.56 ± 3.93	32.20 ± 4.37	0.65
Percent body fat	36.09 ± 4.96	36.90 ± 5.83	0.49
Heart rate (beats/min)	90.00 ± 9.30	80.15 ± 13.02	0.08
SBP (mmHg)	124.62 ± 11.98	125.00 ± 12.25	0.95
DBP (mmHg)	78.46 ± 3.76	76.67 ± 5.16	0.47
TC (mmol/L)	4.93 ± 1.21	5.18 ± 1.36	0.30
HDL (mmol/L)	1.16 ± 0.37	1.31 ± 0.54	0.09
LDL (mmol/L)	3.12 ± 1.08	3.14 ± 1.11	0.93
TG (mmol/L)	2.09 ± 0.36	1.58 ± 1.15	0.20
FBG (mmol/L)	7.69 ± 3.07	7.48 ± 2.86	0.71
HbA1C (DCCT%)	7.23 ± 1.69	7.32 ± 2.15	0.79

All data are presented as the mean ± standard deviation. Abbreviations: BMI, body mass index; SBP, systolic blood pressure; DBP, diastolic blood pressure; TC, total cholesterol; HDL, high-density lipoprotein; LDL, low-density lipoprotein; TG, triglyceride; FBG, fasting blood glucose; HbA1C, glycated haemoglobin

**Table 2:** Characteristics of the T2D subject included in this study according to their Hypertension state

Variables	T2D Hypertension Average ± SD *N* = 49	T2D Non- Hypertension Average ± SD *N* = 29	*p*-value
Age (years)	53.38 ± 11.60	52.37 ± 12.01	0.29
BMI (kg/m2)	32.36 ± 3.87	31.44 ± 3.87	0.31
Percent Body Fat	36.18	36.03 ± 6.31	0.93
Heart Rate	90.00 ± 9.30	84.43 ± 14.57	0.42
SBP (mmHg)	127.14 ± 13.80	125.00 ± 12.25	0.77
DBP (mmHg)	78.57 ± 3.78	76.67 ± 5.16	0.47
TC (mmol/L)	4.89 ± 1.29	5.13 ± 1.60	0.49
HDL (mmol/L)	1.11 ± 0.38	1.28 ± 0.65	0.23
LDL (mmol/L)	3.08 ± 1.50	3.01 ± 1.31	0.82
TG (mmol/L)	2.39 ± 0.51	1.79 ± 1.27	0.29
FBG (mmol/L)	8.50 ± 3.21	8.92 ± 2.94	0.56
HbA1C (DCCT%)	7.83 ± 1.62	8.39 ± 2.17	0.23

All data are presented as the mean ± standard deviation. Abbreviations: BMI, body mass index; SBP, systolic blood pressure; DBP, diastolic blood pressure; TC, total cholesterol; HDL, high-density lipoprotein; LDL, low-density lipoprotein; TG, triglyceride; FBG, fasting blood glucose; HbA1C, glycated haemoglobin

**Table 3:** Characteristics of the non-Diabetic subjects included in this study according to their Hypertension state

Variables	Non-Diabetics Hypertension Average ± SD *N* = 20	Non-Diabetics Non- Hypertension Average ± SD *N* = 21	*p*-value
Age (years)	53.43 ± 10.50	54.00 ± 10.62	0.38
BMI (kg/m2)	33.06 ± 4.14	33.29 ± 4.89	0.87
Percent Body Fat	35.90 ± 5.24	38.24 ± 4.93	0.20
Heart Rate	75.17 ± 9.83	75.25 ± 8.06	0.50
SBP (mmHg)	121.67 ± 10.83	120.02 ± 9.70	0.42
DBP (mmHg)	78.33 ± 4.08	75.40 ± 2.01	0.20
TC (mmol/L)	5.03 ± 0.99	5.25 ± 0.93	0.46
HDL (mmol/L)	1.25 ± 0.33	1.35 ± 0.35	0.35
LDL (mmol/L)	3.22 ± 1.08	3.33 ± 0.74	0.70
TG (mmol/L)	1.36 ± 0.59	1.27 ± 0.88	0.72
FBG (mmol/L)	5.70 ± 1.31	5.44 ± 0.72	0.44
HbA1C (DCCT%)	5.79 ± 0.65	5.73 ± 0.47	0.73
ANGPTL8 pg/ml	1427.18 ± 285.44	949.75 ± 52.80	0.12
ANGPTL4 ng/ml	147.26 ± 12.46	155.39 ± 15.00	0.68
ANGPTL3 ng/ml	69.50 ± 6.37	67.80 ± 5.41	0.84

All data are presented as the mean ± standard deviation. Abbreviations: BMI, body mass index; SBP, systolic blood pressure; DBP, diastolic blood pressure; TC, total cholesterol; HDL, high-density lipoprotein; LDL, low-density lipoprotein; TG, triglyceride; FBG, fasting blood glucose; HbA1C, glycated haemoglobin

### Circulating levels of ANGPTL3, ANGPTL4 and ANGPTL8 in the whole population

**Fig. 1:**
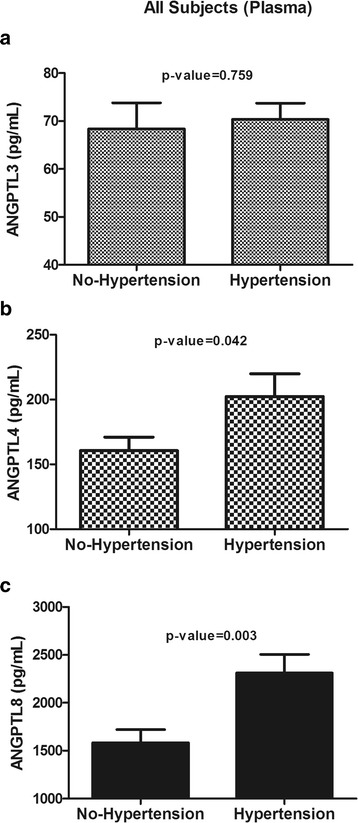
Plasma levels of angiopoietin-like protein 3 (ANGPTL3), ANGPTL4 and ANGPTL8 in the whole population. **a**: Plasma levels of ANGPTL3 in non-hypertensive vs. hypertensive subjects as measured by ELISA. **b**: Plasma levels of ANGPTL4 in non-hypertensive vs. hypertensive subjects as measured by ELISA. **c**: Plasma levels of ANGPTL8 in non-hypertensive vs. hypertensive subjects as measured by ELISA. * *p* < 0.05, as determined using Student’s *t*-test

To examine the circulating levels of the above-mentioned ANGPTLs in subjects with hypertension, we first analysed the differences in their levels between the hypertension and non-hypertension groups. On conducting an analysis of the study population, the circulating levels of ANGPTL4 (202.49 ± 17.44 ng/mL vs. 160.64 ± 10.36 ng/mL, *p* = 0.04) and ANGPTL8 (2310.96 ± 194.88 pg/mL vs. 1583.35 ± 138.27 pg/mL, *p* = 0.001) were higher in subjects with hypertension than in those without hypertension, whereas ANGPTL3 (70.34 ± 3.42 ng/mL vs. 68.35 ± 5.47 ng/mL, *p* = 0.76) levels were not different between the two groups (Fig. 1a, b & c).

### Plasma levels of ANGPTL3, ANGPTL4 and ANGPTL8 in subjects with T2D

**Fig. 2:**
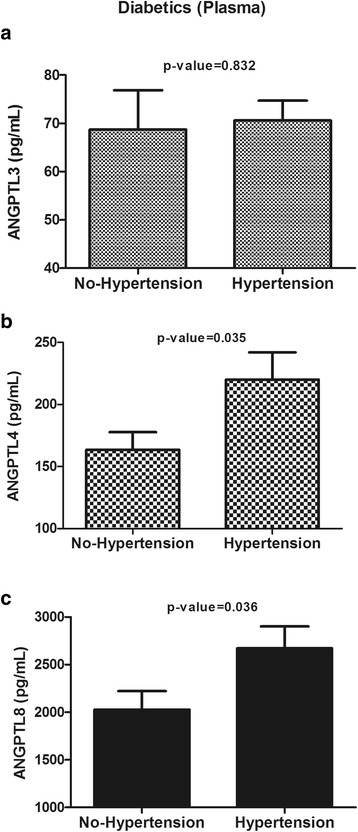
ANGPTL3, ANGPTL4 and ANGPTL8 plasma levels in hypertensive and non-hypertensive type 2 diabetes (T2D) subjects. **a**: Plasma levels of ANGPTL3 in T2D subjects without hypertension vs. those with hypertension as measured by ELISA. **b**: Plasma levels of ANGPTL4 in T2D subjects without hypertension vs. those with hypertension as measured by ELISA. **c**: Plasma levels of ANGPTL8 in T2D subjects without hypertension vs. those with hypertension as measured by ELISA. * *p* < 0.05, as determined using Student’s *t*-test

To investigate the effects of T2D and hypertension on the expression of ANGPTLs, the subjects with T2D were grouped according to their hypertension status. Similar to the entire population of the study, T2D subjects with hypertension had higher circulating levels of ANGPTL4 (220.06 ± 22.06 ng/mL vs. 163.61 ± 14.03 ng/mL, *p* = 0.03) and ANGPTL8 (2671.68 ± 230.75 pg/mL vs. 2026.87 ± 195.50 pg/mL, *p* = 0.04) but not ANGPTL3 (70.63 ± 4.09 ng/mL vs. 68.67 ± 8.20 ng/mL, *p* = 0.83) (Fig. 2a, b & c).

### Plasma levels of ANGPTL3, ANGPTL4 and ANGPTL8 in subjects without T2D

**Fig. 3:**
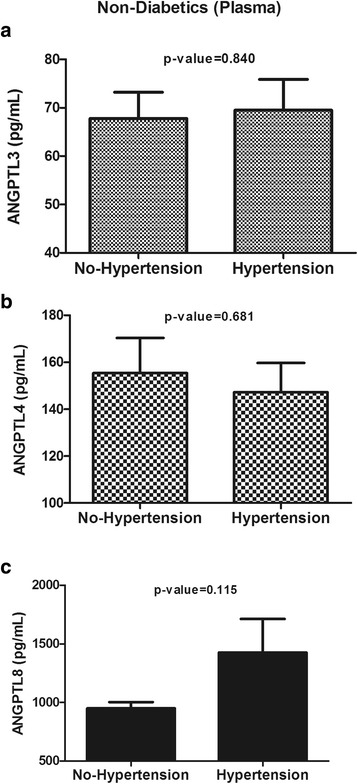
Plasma levels of ANGPTL3, ANGPTL4 and ANGPTL8 in non-diabetic subjects with and without hypertension. **a**: Plasma levels of ANGPTL3 in non-diabetic subjects without hypertension vs. those with hypertension as measured by ELISA. **b**: Plasma levels of ANGPTL4 in non-diabetic subjects without hypertension vs. those with hypertension as measured by ELISA. **c**: Plasma levels of ANGPTL8 in non-diabetic subjects without hypertension vs. those with hypertension as measured by ELISA. * p < 0.05, as determined using Student’s *t*-test

The circulating levels of the above-mentioned ANGPTLs in subjects without T2D according to their hypertension status are presented in Fig. 3a, b & c. The results illustrated that ANGPTL3 (69.50 ± 6.37 ng/mL vs. 67.80 ± 5.41 ng/mL, *p* = 0.84, Fig. 3a), ANGPTL4 (147.26 ± 12.46 ng/mL vs. 155.39 ± 15.00 ng/mL, *p* = 0.68, Fig.e 3b) and ANGPTL8 levels (1427.18 ± 285.44 pg/mL vs. 949.75 ± 52.80 pg/mL, *p* = 0.12, Fig. 3c) were not significantly different in T2D subjects according to the presence or absence of hypertension.

### Multivariable logistic regression analysis for ANGPTL3, ANGPTL4 and ANGPTL8 for predicting hypertension

Multiple logistic regression analysis adjusted showed that subjects in the highest tertile of ANGPTL8 were more likely to have hypertension OR = 3.8, 95% CI = (1.5-9.8), *p*-Value = 0.005. (Table 4). ANGPTL3 and 4 were not significant. Tertile values of ANGPTL3 are expressed as T1 (< 57.83μg/mL), T2 (57.83– 71.55 μg/mL), and T3 (> 71.55 μg/mL). Tertile values for ANGPTL4 are expressed as T1 (< 138.34μg/mL), T2 (138.34 – 190.21 μg/mL), and T3 (> 190.21 μg/mL) and for ANGPTL8 are T1 (< 1263.15 pg/ml), T2 (1263.15-2115.10 pg/ml), and T3 (> 2115.10 pg/ml).

**Table 4:** Multiple logistic regression for hypertension in relation to ANGPTL 3, 4 and 8 as expressed by OR (95% CI)

OR (95% CI)						
	T1	T2	95% CI	T3	95% CI	P-trend
ANGPTL3	1	0.7	(0.3 – 2.0)	0.8	(0.3 - 1.8)	0.525
ANGPTL4	1	1.9	(0.7-5.3)	1.1	(0.5-2.7)	0.228
ANGPTL8	1	3.0	(1.2 -7.6)	3.8	(1.5 – 9.8)	0.005

Multiple logistic regression analysis adjusted for age, gender and BMI. Tertile values of ANGPTL3 are expressed as T1 (< 57.83μg/mL), T2 (57.83– 71.55 μg/mL), and T3 (> 71.55 μg/mL). Tertile values for ANGPTL4 are expressed as T1 (< 138.34μg/mL), T2 (138.34 – 190.21 μg/mL), and T3 (> 190.21 μg/mL) and for ANGPTL8 are T1 (< 1263.15 pg/ml), T2 (1263.15-2115.10 pg/ml), and T3 (> 2115.10 pg/ml)

### Adipose tissue gene expression for ANGPTL3, ANGPTL4 and ANGPTL8

**Fig. 4:**
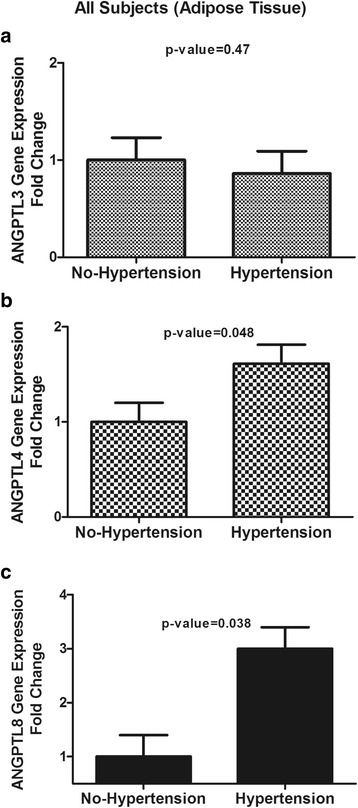
Adipose tissue levels of ANGPTL3, ANGPTL4 and ANGPTL8 in the whole population. **a**: Adipose tissue gene expression of ANGPTL3 in subjects without hypertension vs. those with hypertension in the whole population as measured by real-time PCR. **b**: Adipose tissue gene expression of ANGPTL4 in subjects without hypertension vs. those with hypertension in the whole population as measured by real-time PCR. **c**: Adipose tissue gene expression of ANGPTL8 in subjects without hypertension vs. those with hypertension in the whole population as measured by real-time PCR. * *p* < 0.05, as determined using Student’s *t*-test

**Fig. 5:**
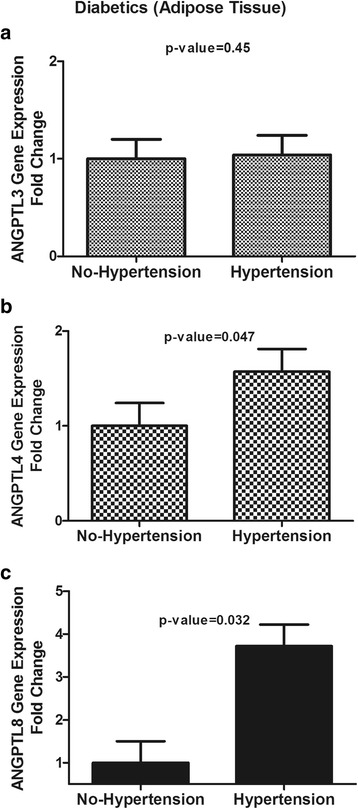
Adipose tissue expression of ANGPTL3, ANGPTL4 and ANGPTL8 in T2D subjects with and without hypertension. **a**: Adipose tissue gene expression of ANGPTL3 in T2D subjects without hypertension vs. those with hypertension as measured by real-time PCR. **b**: Adipose tissue gene expression of ANGPTL4 in T2D subjects without hypertension vs. those with hypertension as measured by real-time PCR. **c**: Adipose tissue gene expression of ANGPTL8 in T2D subjects without hypertension vs. those with hypertension as measured by real-time PCR. * *p* < 0.05, as determined using Student’s *t*-test

To study changes in the expression levels of ANGPTLs in adipose tissue, SAT was obtained from volunteers and gene expression was compared between different groups using real-time PCR. Similar to the plasma levels, ANGPTL3 expression was not different between subjects with and without hypertension (*p* = 0.47, Fig. 4a). Conversely, ANGPTL4 and ANGPTL8 levels were elevated by 1.6- and 3-fold, respectively, in subjects with hypertension compared to those in subjects without hypertension (both *p* < 0.05, Fig. 4b & c). Similarly, in subjects with T2D, ANGPTL4 and ANGPTL8 levels (*p* < 0.05), but not ANGPTL3 levels, were significantly higher in subjects with hypertension (Fig. 5a, b & c).

## Discussion

In this study, we measured the expression of ANGPTL3, ANGPTL4 and ANGPTL8 in both plasma and adipose tissue in subjects with and without hypertension. The circulating levels of ANGPTL4 and ANGPTL8 were increased in subjects with hypertension compared to those in subjects without hypertension in both the entire study population and the T2D subgroup. Conversely, their levels did not differ in subjects without T2D according to their hypertension status. Meanwhile, ANGPTL3 expression did not differ according to the presence of hypertension or diabetes in any of the groups. The gene expression of ANGPTL4 and ANGPTL8 in adipose tissues displayed a similar pattern as the circulating levels in that their expression was elevated in subjects with hypertension. ANGPTL3 levels in adipose tissues did not differ according to the presence or absence of hypertension, as observed for its circulating levels.

The ANGPTL family consists of eight proteins (ANGPTL1–8) that share structural similarities to angiopoietin in that they possess an N-terminal coiled-coil domain as well as a fibrinogen-like domain, except for ANGPTL8, which lacks the fibrinogen-like domain. Some members of this family have been demonstrated to regulate glucose, lipid and energy metabolism. Particularly, ANGPTL3 and ANGPTL4 were revealed to regulate lipid metabolism through their regulation of LPL, the rate-limiting enzyme in lipoprotein hydrolysis. ANGPTL8 has also been found to play a similar role in regulating LPL through its interaction with ANGPTL3. ANGPTL4 has been investigated as a therapeutic target based on its role in regulating lipid levels in plasma. Many studies illustrated that loss-of-function mutations in ANGPTL4, particularly the E40K substitution, are associated with reduced plasma levels of TG and HDL [12, 21–23]. Recently reported data indicated that subjects carrying the E40K substitution had a significantly lower risk of coronary artery disease than non-carriers [12]. Furthermore, prior research uncovered that mice injected with ANGPTL4 monoclonal antibodies exhibited low plasma TG levels as well as increased LPL activity [24]. Considering these data, the increased levels of ANGPTL4 in both plasma and adipose tissues of subjects with hypertension in our study support that this protein may participate in hypertension development in this population. This could occur through the activation of ANGPTL4 by hypoxia, which is a known inducer of ANGPTL4 expression [25, 26].

ANGPTL8 has been recognised as a dual-role protein that regulates lipid metabolism via regulation of LPL. Our group and others recently demonstrated that ANGPTL8 levels were increased in T2D as well as obesity, suggesting a pathogenic role for this protein in these diseases [14, 15]. It has been found that ANGPTL8 overexpression leads to increased TG plasma levels [11, 27, 28]. This increase was dependant on an interaction with ANGPTL3, as ANGPTL8 overexpression did not result in increased TG levels in mice lacking ANGPTL3 [11]. However, ANGPTL3 overexpression alone did not cause an increase in TG levels, suggesting that ANGPTL8 was the limiting factor. In support of this argument, it has been found that in mice, ANGPTL3 was present in excess and ANGPTL8 expression is the rate-limiting step [11]. This could explain our present data and the lack of changes in ANGPTL3 levels in hypertensive subjects. In light of these data, ANGPTL8 could be a more effective therapeutic target than ANGPTL3.

One of the main limitations of the study is its cross-sectional design, which did not allow us to establish the causality and role that ANGPTLs may play in hypertension development. However, the atherogenic roles of these proteins are inferred from their function and previous findings regarding their roles in increasing plasma lipid content. Nonetheless, a prospective study will more accurately answer this question and establish their roles in increasing the incidence of hypertension as well as cardiovascular risks.

## Conclusion

In conclusion, ANGPTL4 and ANGPTL8 levels are increased in both plasma and adipose tissues of subjects with hypertension. This novel finding highlights the atherogenic nature of these proteins and their potential contributions to increased cardiovascular risks. Conversely, ANGPTL3 levels did not differ according to the presence or absence of hypertension, suggesting that ANGPTL3 may not be as effective a therapeutic target for reducing cardiovascular risks as ANGPTL4 and ANGPTL8.

## Abbreviations

ANGPTL: Angiopoietin-like protein
BMI: Body mass index
CV: Coefficients of variation
DBP: Diastolic blood pressure
ELISA: Enzyme-linked immunosorbent assay
FBG: Fasting blood glucose
HbA1C: Glycated haemoglobin
HDL: High-density lipoprotein
HsCRP: High-sensitivity C-reactive protein
LDL: Low-density lipoprotein
PCR: Polymerase chain reaction
SAT: Subcutaneous adipose tissue
SBP: Systolic blood pressure
TC: Total cholesterol
TG: Triglyceride
